# Factors Associated With Prescriptions for Branded Medications in the Medicare Part D Program

**DOI:** 10.1001/jamanetworkopen.2021.0483

**Published:** 2021-03-02

**Authors:** Mariana P. Socal, Ge Bai, Gerard F. Anderson

**Affiliations:** 1 Department of Health Policy and Management, Johns Hopkins Bloomberg School of Public Health, Baltimore, Maryland; 2 Johns Hopkins Carey Business School, Washington, DC; 3 Department of Health Policy and Management, Johns Hopkins Bloomberg School of Public Health, Washington, DC; 4 Department of Health Policy and Management, Department of International Health, Johns Hopkins Bloomberg School of Public Health, Johns Hopkins University School of Medicine, Baltimore, Maryland

## Abstract

**Importance:**

Branded products of multisource drugs are frequently dispensed in the Medicare Part D program, increasing costs for the program and patients.

**Objective:**

To examine the reasons for dispensing branded multisource drugs in Medicare Part D.

**Design, Setting, and Participants:**

This cross-sectional study examined claims for multisource drugs with more than 1000 branded claims dispensed in Medicare Part D using Medicare Prescription Drug Event data from a 2017 nationwide random sample of 20% of Medicare beneficiaries. Data were analyzed between January and October 2020.

**Exposures:**

Justification for branded dispensing as indicated by each claim’s dispense-as-written code.

**Main Outcomes and Measures:**

Mean Medicare Part D program spending and patient out-of-pocket spending for branded and generic products, and generic vs branded spending discounts in program and patient out-of-pocket spending for each multisource drug.

**Results:**

Among 169 million claims for 224 multisource drugs, 8.3 million claims (4.9%) were dispensed with a branded product. Among these claims, 4.9 million claims (59.2%) did not have a recorded reason for branded dispensing; 1.4 million claims (16.9%) occurred because of prescriber requests; and 1.1 million claims (13.5%) occurred because of patient requests. If all branded dispensing requested by prescribers had been substituted by the corresponding generics, the projected savings to the Medicare Part D program and Medicare patients were $997 million (56.0%) and $161 million (64.4%), respectively. If all branded dispensing requested by patients had been substituted by generics, the projected savings to the Medicare Part D program and Medicare patient were $673 million (53.4%) and $109 million (55.1%), respectively. Drugs with the highest proportion of branded dispensing by physician or patient request were typically high cost (eg, drugs with above-median frequencies of branded dispensing: mean [SD] discount on generic vs branded, 73.9% [26.9%] for prescriber requests).

**Conclusions and Relevance:**

Prescribers and patients motivated 30.4% of all branded dispensing of multisource drugs in the Medicare Part D program. Branded dispensing requested by prescribers or patients incurred an incremental annual cost of $1.67 billion to the Medicare program and $270 million to patients when compared with switching to generics. Policy makers should consider ways to discourage prescribers and patients from requesting branded dispensing of multisource drugs because of the higher cost.

## Key Points

**Question:**

What factors are associated with the dispensing of branded medications in the Medicare Part D program?

**Findings:**

This cross-sectional study of 169 million Medicare part D multisource prescription drug claims in 2017 revealed that branded drugs were dispensed because of prescriber request 16.9% of the time and because of patient request 13.5% of the time. The projected savings for switching the branded drugs requested by prescribers to generics were $997 million for Medicare and $161 million for patients; projected savings for switching patient-requested drugs were $673 million for Medicare and $109 million for patients.

**Meaning:**

Branded dispensing of multisource drugs requested by prescribers or patients was associated with increased spending for the Medicare program and patients.

## Teaser

This cross-sectional study assessed factors associated with prescriptions of branded medications when generic drugs were available using Medicare part D multisource prescription drug claims in 2017.

## Introduction

Promoting the use of generics—the lower-priced and therapeutically equivalent versions of branded drugs—is an important approach to contain health care spending in the US.^1,2,3,4,5^ The US Department of Health and Human Services estimated that, if all branded drugs with generic equivalents had been dispensed with a generic in the Medicare Part D program, the program and its beneficiaries would have saved $2.8 billion in 2016.^6^ Since 1984, all states and the District of Columbia have enacted laws to promote generic dispensing, either through mandatory substitution (pharmacists must dispense generics when filling a branded prescription) or permissive substitution (pharmacists have the option to dispense generics).^4,7,8^ However, the instruction of prescribers, the preference of patients, and other reasons such as the unavailability of a generic may override state substitution laws.^4,8,9^ The specific reason for dispensing a given product is documented through product selection codes, also known as dispense-as-written (DAW) provisions (Table 1). Physicians may indicate DAW provisions on their prescriptions or pharmacists may document such provisions when they file the drug claim.^10^

**Table 1.** Description of Dispense-as-Written (DAW) Codes^a^

Code	Description
0	No product selection indicated (may also have missing values)
1	Substitution not allowed by prescriber (branded product requested by the prescriber)
2	Substitution allowed—patient requested that brand product be dispensed
3	Substitution allowed—pharmacist selected product dispensed
4	Substitution allowed—generic drug not in stock
5	Substitution allowed—brand drug dispensed as generic
6	Override
7	Substitution not allowed—brand drug mandated by law
8	Substitution allowed—generic drug not available in marketplace
9	Other

^a^ Table was adapted from the Chronic Conditions Data Warehouse CCW Medicare Part D Data User Guide, October 2019.^14^ DAW codes do not necessarily represent how the drug was actually dispensed.

There is limited research on DAW provisions. A 2009 study^4^ of employer-sponsored health insurance plans managed by a large pharmaceutical benefits manager found that, among a total of 5.6 million prescriptions, 2.7% were designated as DAW 1 (branded product requested by the prescriber) and 2% were designated as DAW 2 (branded product requested by the patient). A 2018 study^9^ examined a single drug—imatinib mesylate, an oral agent used to treat leukemia—and found that 24% of all imatinib claims dispensed with the branded product in commercial insurance plans between 2001 and 2017 had been recorded as requested by the physician (DAW 1) or the patient (DAW 2). These studies, limited to a single drug or a single pharmaceutical benefits manager, are not readily generalizable to the Medicare Part D program. Medicare accounts for approximately one-third of the total prescription drug spending in the US.^11^

To date, the circumstances regarding dispensing of branded multisource drugs in the Medicare Part D program have received limited attention in spite of the cost-saving potential of generic substitution as incentivized by state generic substitution laws. This question has strong policy relevance given the substantial price differential between most branded and generic products and the recent focus by lawmakers on controlling drug spending.^12^ In this study, we used a nationwide random sample of 20% of Medicare beneficiaries to examine DAW provisions among 169 million claims for multisource drugs dispensed in the Medicare Part D program in 2017.^13^

## Methods

### Data and Sample

This study was approved by the institutional review board at the Johns Hopkins Bloomberg School of Public Health. This study follows the Consolidated Health Economic Evaluation Reporting Standards (CHEERS) reporting guideline for economic evaluations of health interventions.

We examined DAW provisions in Medicare Part D pharmaceutical claims data using the 2017 Medicare Part D Prescription Drug Event files from a 20% nationwide random sample of Medicare beneficiaries.^13,14^ Drugs not covered by Medicare Part D and nondrug products, such as syringes and antiseptics, were excluded. Overall, there were 299 million pharmaceutical claims recorded among this sample in 2017, representing a total of 2398 different drugs. A drug was defined by its active ingredient, route of administration, and dosage form; products were compared without accounting for possible differences in strength.

A total of 754 drugs (31.4%) were determined to be multisource because they had at least 1 branded and 1 generic product dispensed in Medicare Part D in 2017. Of those, 224 drugs had at least 1000 claims dispensed with the branded product(s) in our data and constituted the final sample of this study. There were 169 million claims for these 224 drugs, representing 56.7% of all Part D drug claims in 2017. Because a drug can have multiple generic and branded products, the 224 drugs represented a total of 635 different products (227 generic and 408 branded).

### Variable Measurement

From each claim, we collected information on the type of product dispensed (ie, branded or generic), the Medicare Part D program spending, and the patient out-of-pocket spending. All claims were accompanied by a DAW code indicating the recorded reason for dispensing the product (Table 1). No claim had a missing DAW code. The program spending information in this study accounted for any upfront discounts provided by drug manufacturers to Medicare Part D prescription drug plans. However, rebate information was not available and, therefore, rebates were not incorporated in the analysis.

For each drug in the sample, we calculated the proportion of branded claims and the frequency of branded dispensing by each DAW code (ie, number of branded claims dispensed with the DAW code divided by the drug's total number of claims dispensed with a branded product).

### Statistical Analysis

Data were analyzed between January and October 2020. We conducted descriptive analyses to examine the frequency of branded dispensing associated with each DAW code. For each of the 3 most common codes (DAW 0, no product selection indicated; DAW 1, branded product requested by the physician; and DAW 2, branded product requested by the patient), we calculated the total actual program spending and patient out-of-pocket spending on branded claims as well as the total projected program spending and patient out-of-pocket spending assuming all branded claims had been dispensed with a generic. For the calculation of the projected spending, we used the weighted average program spending and the weighted average patient out-of-pocket spending per claim for the corresponding generic, with the number of generic claims as the weighting factor. Since the sample consisted 20% of Medicare beneficiaries, we multiplied all actual and projected spending amounts by a factor of 5 to extrapolate the amounts pertaining to the entirety of the Medicare Part D program and its patients. Weighting was necessary because some products had greater dispensing volume than others.

Next, we ranked all drugs by their frequency of branded claims dispensed under each of the 3 most common DAW codes (DAW 0, DAW 1, and DAW 2). For each DAW code, we focused on the drugs above the median of branded dispensing recorded with that code and calculated the mean program and patient out-of-pocket generic spending discount among these drugs. The generic spending discount reflects the percentage of savings provided by generics relative to branded products for the drug. The discount was measured as the difference between the drug’s weighted average spending per claim on the branded products (in which the weighting factor is the number of branded claims) and the weighted average spending per claim on the generic products (with the weighting factor being the number of generic claims), divided by the drug’s weighted average spending per claim on the branded products.

Lastly, we identified the top 10 drugs with the highest frequency of branded dispensing with DAW 0, DAW 1, and DAW 2, respectively. For each drug, we examined the number of branded products available on the market, and the weighted average Medicare Part D spending and patient out-of-pocket spending corresponding to the drug’s branded and generic products. Analysis was conducted using STATA version 16.1 (StataCorp).

## Results

### Drug Dispensing Overview

**Figure 1.**
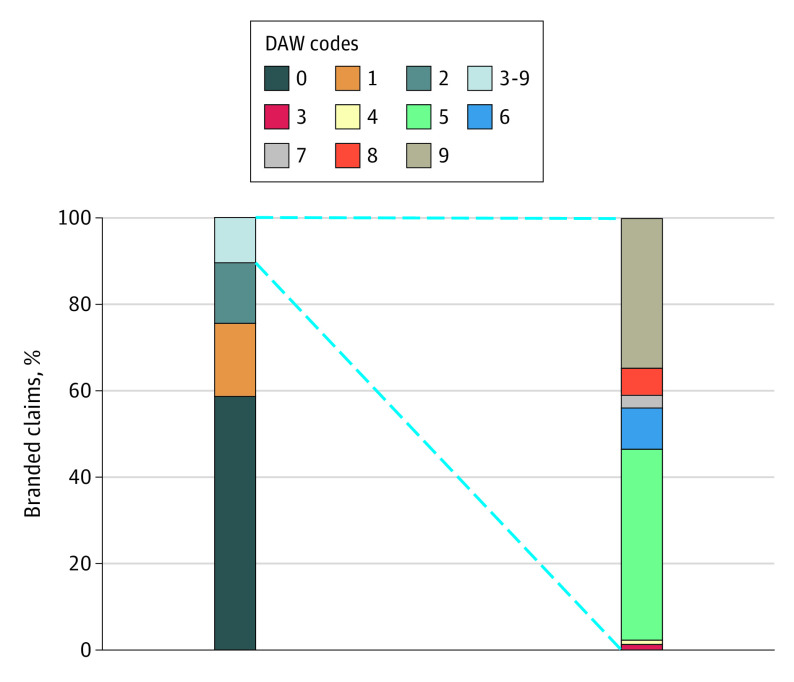
Dispense-as-Written (DAW) Codes Recorded Among Branded Dispensing of Multisource Drugs in the Medicare Part D Program Data total 8.3 million claims representing 408 different multisource branded products obtained from Medicare Part D Prescription Drug Event files, Chronic Conditions Data Warehouse, 2017.^13,14^

Of the 169 million claims for the 224 multisource drugs in the study sample, 161 million claims (95.1%) were dispensed with a generic product and 8.3 million claims (4.9%) were dispensed with a branded product. Among the claims dispensed with a branded product, 4.9 million claims (59.2%) had no product selection indicated (DAW 0); 1.4 million claims (16.9%) indicated that the branded product was requested by the physician (DAW 1); 1.1 million claims (13.5%) indicated that the branded product was requested by the patient (DAW 2); and the remaining 0.9 million claims (10.4%) had a mix of other DAW provisions (Figure 1). All drugs in the sample had at least 1 branded claim dispensed under codes DAW 0, DAW 1, and DAW 2.

### Medicare Part D Program and Patient Out-of-Pocket Spending

**Figure 2.**
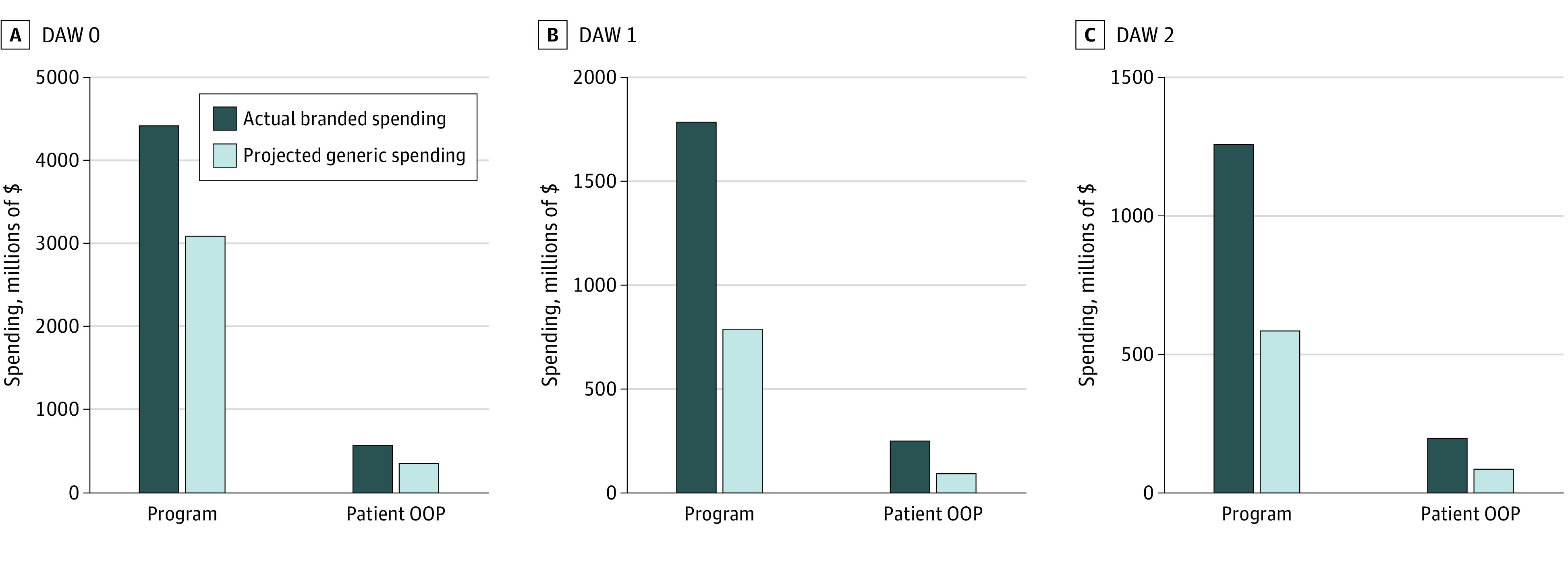
Spending on Branded Drugs vs Projected Spending Under Generic Substitution, by Dispense-as-Written (DAW) Code DAW 0 indicates no product selection indicated; DAW 1, branded product requested by the prescriber; DAW 2, patient requested that brand product be dispensed; OOP, out-of-pocket. Analysis of Medicare Part D Prescription Drug Event files for a 20% nationally random sample of Medicare beneficiaries, a total of 169 million claims representing 224 multisource drugs. Actual and projected spending values from the 20% random sample were multiplied by a factor of 5. Data obtained from the Medicare Part D Prescription Drug Event files, Chronic Conditions Data Warehouse, 2017.^13,14^

In 2017, the Medicare Part D program spent a total of $4.42 billion on claims dispensed with a branded product where no product selection was indicated (DAW 0) (Figure 2); patients spent a total of $589 million out-of-pocket on these drugs. On branded drugs dispensed at the prescriber’s request (DAW 1), the Medicare Part D program spent a total of $1.78 billion and patients spent a total of $250 million out-of-pocket in the same period. The Medicare Part D program spent a total of $1.26 billion and patients spent a total of $198 million on branded drugs dispensed at the patient’s request (DAW 2).

Under full generic substitution, claims dispensed with DAW 0 would have cost the Medicare Part D program a total of $3.10 billion in 2017 (saving $1.33 billion, or 30.1%). Such claims would have cost patients a total of $368 million out-of-pocket (saving $221 million, or 37.5%). Claims dispensed with DAW 1 would have cost the Medicare Part D program a total of $787 million (saving $997 million, or 56.0%) and patients a total of $89 million out-of-pocket (saving $161 million, or 64.4%). Claims dispensed with DAW 2 would have cost the Medicare Part D program a total of $586 million (saving $673 million, or 53.4%) and patients a total of $89 million out-of-pocket (saving $109 million, or 55.1%). Taken together, projected savings for full generic substitution of all branded drugs dispensed under DAW 1 and DAW 2 were $1.67 billion to the Medicare Part D program and $270 million to Medicare patients in 2017.

### Generic vs Branded Spending Discounts

**Figure 3.**
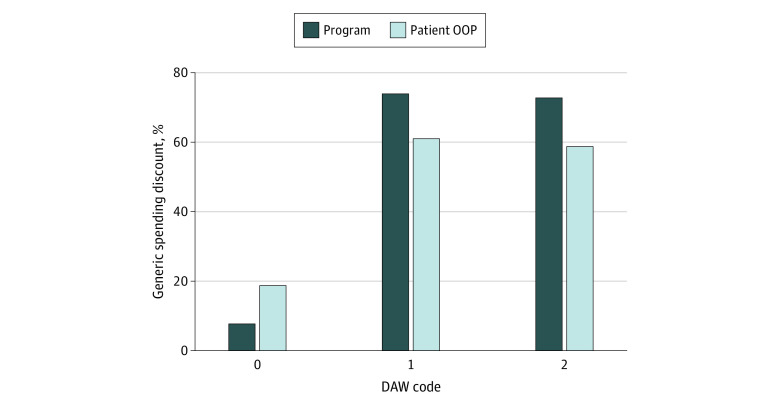
Mean Branded vs Generic Spending Discount for Drugs Above the Median Frequency of Dispense-as-Written (DAW) Provisions DAW 0 indicates no product selection indicated; DAW 1, branded product requested by the prescriber; DAW 2, patient requested that brand product be dispensed; OOP, out-of-pocket. Analysis of Medicare Part D Prescription Drug Event files for a 20% nationally random sample of Medicare beneficiaries, a total of 169 million claims representing 224 different multisource drugs. All drugs had at least 1 claim with DAW 0, DAW 1, and DAW 2 codes. For drugs with frequency of DAW dispensing above the median, each drug contributed its mean spending on branded and generic products, weighted by the corresponding number of branded or generic claims. Data obtained from the Medicare Part D Prescription Drug Event files, Chronic Conditions Data Warehouse, 2017.

Among drugs with above-median frequency of branded dispensing and with no product selection indicated (DAW 0), the mean (SD) Medicare Part D program generic spending discount was 7.8% (81.3%) (median [interquartile range {IQR}], 16.3% [0.9%-42.9%]), indicating that the program spent on average 7.8% more on branded than on generic claims (Figure 3). The mean (SD) patient out-of-pocket generic spending discount was 19.0% (60.1%) (median [IQR], 26.5% [5.2%-50.7%]), indicating that patients spent on average 19.0% more on branded than on generic claims. Among drugs with above-median frequency of branded dispensing requested by the prescriber (DAW 1), the mean (SD) program generic spending discount was 73.9% (26.9%) (median [IQR], 86.0% [52.9%-97.0%]), and the mean (SD) patient out-of-pocket generic spending discount was 61.1% (32.7%) (median [IQR], 67.5% [45.8%-86.9%]). Among drugs with above-median branded dispensing requested by the patient (DAW 2), the mean (SD) program generic spending discount was 72.8% (28.6%) (median [IQR], 86.3% [51.1%-97.0%]), and the mean (SD) patient out-of-pocket generic spending discount was 58.9% (35.2%) (median [IQR], 67.3% [39.8%-87.1%]).

### Drugs With the Highest Frequency of DAW 0, DAW 1, and DAW 2 Branded Dispensing

The top 10 drugs with the highest frequency of branded dispensing requested by the prescriber (DAW 1) or the patient (DAW 2) had more expensive branded products than the top 10 drugs with the highest frequency of branded dispensing with no product selection indicated (DAW 0) (Table 2). While the unit cost of branded products in the top 10 DAW 0 drugs ranged between $2.88 and $27.72, the unit cost of branded products in the top 10 DAW 1 drugs ranged between $306.21 and $14 671.90, and the unit cost of branded products in the top 10 DAW 2 drugs ranged between $122.43 and $517.22. Moreover, 9 of the top 10 DAW 1 drugs and all top 10 DAW 2 drugs had only 1 branded product available on the market. In contrast, 3 of the top 10 DAW 0 drugs had a single branded product available on the market.

**Table 2.** Top 10 Drugs With the Highest Frequency of Branded Dispensing by Dispense-as-Written (DAW) Code, 2017

Generic name	Dosage form description	Brand name(s)	Main use or indication	Claims, %		Spending, $^c^			
				Branded^a^	DAW^b^	Program		OOP	
						Branded	Generic	Branded	Generic
**DAW 0 (No product selection indicated)**									
Ibuprofen	Tablets	Ibu	Anti-inflammatory	25.6	99.8	2.88	2.64	2.00	2.11
Potassium chloride	Capsule extended release, sprinkle	Klor-Con Sprinkle	Mineral supplement	21.4	99.4	16.58	15.73	7.35	9.41
Lactulose	Oral solution	Constulose, Generlac, Enulose	Laxative	19.3	99.3	9.62	8.88	3.09	2.79
Prenatal vitamins, calcium 72/iron/folic acid	Tablets	Preplus, Prenatal Plus	Vitamins	86.1	99.3	5.68	3.47	2.92	2.41
Nystatin	External powder	Nyamyc, Nystop	Antifungal	69.8	99.2	15.21	16.10	5.53	5.60
Diltiazem HCL	Capsule extended release 24 Hour	Cardizem CD, Cartia XT, Dilt-CD	Angina and hypertension	41.3	98.9	14.58	22.56	13.57	13.29
Diltiazem HCL	Extended release capsules	Dilt-XR	Angina and hypertension	71.0	98.9	17.19	13.51	15.77	14.09
Potassium chloride	Tablet extended release	Klor-Con M20, M15, M10	Mineral supplement	19.3	98.9	11.26	11.23	9.74	6.27
Gentamicin sulfate	Ophtalmic ointment	Gentak	Antibacterial	32.8	98.5	5.26	34.15	5.69	10.08
Hydrocortisone	Rectal cream	Proctosol-HC, Proctozone-HC, Procto-Med HC, Anusol-HC, Procto-Pak	Hemorrhoids	92.7	98.4	27.72	26.19	8.12	7.91
Median (IQR) for top 10 drugs	NA	NA	NA	37.1 (20.9-74.8)	99.1 (8.8-99.3)	12.92 (5.58-16.73)	14.62 (7.53-23.47)	6.52 (3.05-10.70)	7.09 (2.70-10.88)
**DAW 1 (branded product requested by the prescriber)**									
Tetrabenazine	Tablets	Xenazine	Huntington's chorea	27.7	93.0	14 671.90	4852.63	465.85	147.30
Lamotrigine	Tablet extended release 24 Hour	Lamictal XR	Epilepsy	18.3	83.9	1454.14	314.09	37.39	22.91
Zonisamide	Capsule	Zonegran	Epilepsy	2.4	83.1	1919.43	13.13	19.07	4.56
Lamotrigine	Tablet	Lamictal	Epilepsy	2.0	81.1	1048.41	4.92	27.69	3.27
Divalproex sodium	Tablet delayed release	Depakote	Epilepsy	1.3	80.8	359.86	9.70	19.94	2.91
Levetiracetam	Tablet	Keppra, Roweepra	Epilepsy	1.7	79.1	914.90	10.52	32.62	4.94
Divalproex sodium	Tablet extended release 24 Hour	Depakote ER	Epilepsy	2.1	78.7	306.21	62.44	18.17	6.08
Tacrolimus	Capsule	Prograf	Immunosuppression post–organ transplantation	6.4	78.3	416.16	87.25	56.72	21.51
Levetiracetam	Tablet extended release 24 Hour	Keppra XR	Epilepsy	14.0	78.2	831.36	34.28	41.30	15.72
Oxcarbazepine	Tablet	Trileptal	Epilepsy	1.5	77.1	789.52	15.10	29.77	7.21
Median (IQR) for top 10 drugs	NA	NA	NA	2.3 (1.7-15.1)	80.0 (78.3-83.3)	873.13 (402.09-1570.46)	24.69 (10.32-143.96)	31.20 (19.72-45.16)	6.65 (4.24-21.86)
**DAW 2 (branded product requested by the patient)**									
Amlodipine/atorvastatin	Tablet	Caduet	Hypertension and high cholesterol	4.5	54.8	399.00	166.13	39.03	29.35
Dofetilide	Capsule	Tikosyn	Antiarrhythmic	10.3	51.2	375.42	292.38	132.26	102.12
Amlodipine besilate/olmesartan medoxomil	Tablet	Azor	Hypertension	14.7	51.2	219.35	89.44	50.94	23.11
Dutasteride/tamsulosin HCL	Capsule	Jalyn	Benign prostatic hyperplasia	6.4	48.0	133.00	105.88	61.05	35.96
Olmesartan/amlodipine/ hydrochlorothiazide	Tablet	Tribenzor	Hypertension	16.6	47.2	219.58	135.45	44.79	29.84
Doxazosin mesylate	Tablet	Cardura	Benign prostatic hyperplasia	0.2	46.3	122.43	12.00	30.34	10.04
Armodafinil	Tablet	Nuvigil	Sleep apnea, narcolepsy	16.1	46.3	517.22	131.47	47.30	24.39
Olmesartan/hydrochlorothiazide	Tablet	Benicar HCT	Hypertension	25.5	46.1	242.67	100.41	48.58	32.93
Eszopiclone	Tablet	Lunesta	Sedative/ hypnotic	2.9	45.3	292.89	17.56	44.62	14.43
Olmesartan medoxomil	Tablet	Benicar	Hypertension	23.6	45.3	230.30	93.21	48.04	31.91
Median (IQR) for top 10 drugs	NA	NA	NA	12.5 (4.1-18.4)	46.8 (45.9-51.2)	236.49 (197.76-381.32)	103.15 (71.47-143.12)	47.67 (43.22-53.47)	29.60 (20.94-33.69)

Abbreviations: NA, not applicable; OOP, out-of-pocket.

^a^ Indicates the frequency of branded dispensing among all dispensing of the drug.

^b^ Indicates the frequency of branded dispensing designated with that DAW code among all branded dispensing of the drug.

^c^ Medicare Part D Program spending and patient OOP spending are calculated as the mean spending on branded or generic claims of the drug, weighted by the number of claims for the corresponding branded or generic products of that drug.

Comparing the top 10 drugs across the 3 DAW provisions, we found that the top 10 DAW 0 drugs mostly reflected branded versions of over-the-counter products. These drugs were affordable regardless of being a branded or generic version, and their branded vs generic price differentials were minimal. Therefore, patients and pharmacies may have little reason to prefer one product over the other. All top 10 DAW 1 drugs were antiepileptic drugs—a therapeutic class where switching between products with any potential differences in bioavailability is generally avoided, as it may result in toxicity or loss of seizure control.^15^ The top 10 DAW 2 drugs reflected some combination medications, often for the treatment of high blood pressure, for which combination generics are also available.

## Discussion

Based on the 20% Medicare sample in 2017, we found that among 169 million claims for 224 multisource drugs, which represented 56.7% of all claims for covered drugs dispensed in the Medicare Part D program, 8.3 million claims (4.9%) were dispensed with a branded product. Of these 8.3 million claims, 16.9% occurred because of prescriber requests (DAW 1) and 13.5% occurred because of patient requests (DAW 2). Had they been dispensed with the corresponding generic, these claims would have generated a projected $1.67 billion savings ($997 million for DAW 1 and $673 million for DAW 2) to the Medicare Part D program and $270 million out-of-pocket savings ($161 million for DAW 1 and $109 million for DAW 2) for Medicare patients. Our annual projected savings from full generic substitution of all branded drugs dispensed under DAW 0, DAW 1, and DAW 2 ($3.00 billion for the Medicare program and $491 million for patients) were consistent with previous findings from the Office of the Assistant Secretary for Planning and Evaluation of the US Department of Health and Human Services.^6^

Drugs with the higher than median frequency of DAW 1 and DAW 2 branded dispensing had substantially more expensive branded products than generic products. For DAW 1, switching the branded products of these drugs to generics would have generated on average 73.9% savings to the Medicare Part D program and 61.1% out-of-pocket savings to patients in 2017. For branded dispensing coded DAW 2, switching the branded products of these drugs to generics would have generated on average 72.8% savings to the Medicare Part D program and 58.9% out-of-pocket savings to patients.

Most top 10 drugs with the highest frequency of DAW 1 or DAW 2 branded dispensing had only 1 branded product on the market, suggesting that the marketing strategies for branded drugs without other branded competitors might be more influential over prescriber and/or patient preferences as compared with the marketing strategies of drugs with multiple branded competitors. In addition, most top 10 DAW 1 drugs being antiepileptic drugs with clinical nuances indicates prescribers’ discretion in choosing what specific product to be dispensed; some top 10 DAW 2 drugs being combination medications suggests that patients may prefer taking 1 pill instead of multiple pills. However, all of these drugs had combination generics available, and research has shown that patients’ willingness to pay to reduce the number of tablets in each dose is very limited.^16^

DAW 0 (“No product selection indicated”) accounted for 59.2% of all branded claims of multisource drugs. DAW 0 is the default selection in any pharmacy system and indicates that the prescriber, patient, or third-party payer did not specify that a specific product must be dispensed. The findings that branded dispensing under DAW 0 did not generate substantial inefficiency suggest that pharmacies may have chosen to dispense branded drugs when their prices were comparable with the prices of the available generic substitutes.

DAW 1 (“Requests from prescribers”) represented the largest proportion of branded dispensing recorded with a specified provision, and frequently occurred for drugs with large generic vs branded price differentials. Therefore, policies targeted on prescriber behavior are likely to have the greatest potential to promote generic use and generate savings. Prescribing physicians have substantial influence over patients’ medication preferences.^17,18^ Multiple surveys have found that skepticism about generic medications is common among physicians, especially among physicians who have more exposure to pharmaceutical marketing activities.^2,18^ Efforts to improve physicians’ perception of generic medications, raising their awareness of the availability of generics, requiring them to write a phrase rather than check a DAW box when issuing the prescription, and limiting their exposure to pharmaceutical marketing might be effective in enhancing generic use.^2,4,18,19^ Currently, the lack of awareness of price differences at the point of care prevents prescribers from identifying the most affordable product, creating a potential barrier to patients’ drug adherence. Point-of-care resources, such as access to electronic price databases, would also allow prescribers to compare prices between multiple product options so as to make more informed decisions. In addition, the US Centers for Medicare & Medicaid Services could consider sharing individualized, confidential report cards to physicians with high DAW requests. Informing outlier prescribers of their relative standing as compared with their peers may help mitigate their requests for branded drugs.^20,21^

Patients’ requests for branded products (DAW 2) constitute another barrier to greater generic use. Drugs with high frequency of DAW 2 dispensing had large generic vs branded price differentials. A recent national survey found that 37% of patients prefer branded products to generics, and 46% of patients asked their physicians to prescribe a brand-name product rather than a generic at least once in the past year.^22^ Medicare beneficiaries tend to have multiple chronic conditions and many have difficulty affording treatments. Medicare beneficiaries’ drug choices and use also have implications for Medicare program spending and taxpayer burden. At the very least, patients should be made aware of the extra costs associated with branded drug use in order to make more informed choices. Limiting direct-to-consumer advertising and educating patients on the safety and effectiveness of generic drugs have been suggested as options to improve patient perception.^4,22^ Improving formulary management and pharmacy benefit design can further incentivize patients to use generics.^17,23,24^ Centers for Medicare & Medicaid Services could also consider communicating to beneficiaries the lower out-of-pocket costs and the potential savings that could be generated for the Medicare Part D program from generic substitution to influence beneficiary preferences.

It is worth noting that some patients may have preferences for branded drugs over generics.^16,22,25^ In this case, DAW 1 and DAW 2 dispensing would enable these patients to obtain benefits from taking branded drugs. If patients had fully absorbed the costs of such branded drugs, then DAW 1 and DAW 2 dispensing would not have raised concerns because the dispensing decision would reflect the patient’s utility function. However, in the Medicare Part D program, dispensing decisions have implications for overall program spending, and the financial implications from inefficient dispensing are shared between the patient and the program. Although branded dispensing accounted for only 5% of multisource drug claims, it incurred $3 billion incremental spending each year to the program and almost $500 million for patients. A feasible policy goal would be not to eliminate, but mitigate inefficient branded dispensing of multisource drugs. Understanding the financial implications of branded dispensing can facilitate cost containment in the Medicare Part D program.

### Limitations

This study has several limitations. First, a multisource drug was defined without accounting for strength. If, for the same drug, branded products had substantially greater or lower number of claims for a specific strength than generics, our results could have been skewed. Because we conducted our analysis at the claim level, our results may also have been skewed if branded claims had systematically greater or lower number of units per claim than generics. Second, we identified whether a product was generic or branded by comparing its proprietary name with its nonproprietary name. This approach may have misclassified a trademarked generic as a branded product, and thus our study could have underestimated the branded vs generic price differentials. Third, this study only examined multisource drugs with more than 1000 branded claims. Although these drugs represented 56.7% of Medicare Part D drug claims in 2017, our findings may not be generalizable to all multisource drugs in the US market. Fourth, drug rebate information was not available, which may have increased our estimated program spending differentials between branded and generic products. However, out-of-pocket spending comparisons would be unchanged. Lastly, patient preferences and physician preferences might interact, and thus it is likely that branded claims designated with DAW 1 and DAW 2 should not be considered in isolation from each other.

## Conclusions

Prescribers and patients’ requests accounted for 30.4% of all branded dispensing of multisource drugs in the Medicare Part D program. Branded drugs frequently requested by prescribers or patients were expensive and had substantially more affordable generics, thus generating increased spending for the Medicare program and beneficiaries. Policy initiatives aimed at containing prescription drug spending should consider discouraging prescribers and patients from requesting branded dispensing.
